# Association of Serial Ventricular Routine Cerebrospinal Fluid Findings with Complications and Outcome in Aneurysmal Subarachnoid Hemorrhage: A Retrospective Observational Study

**DOI:** 10.1007/s12028-025-02406-x

**Published:** 2025-11-14

**Authors:** Claudio Togni, Mina Pasqualini, Ignazio de Trizio, Francesca Casagrande, Federica Stretti, Emanuela Keller, Giovanna Brandi

**Affiliations:** 1 Department of Neurology, Clinical Neuroscience Center, University Hospital Zurich, University of Zurich, Zurich, Switzerland; 2 Institute of Intensive Care Medicine, University Hospital Zurich, University of Zurich, Zurich, Switzerland; 3 Department of Neurosurgery, Clinical Neuroscience Center, University Hospital Zurich, University of Zurich, Zurich, Switzerland

**Keywords:** Cerebrospinal fluid, Subarachnoid hemorrhage, Delayed cerebral ischemia, Hydrocephalus, Functional neurological outcome

## Abstract

**Background:**

Cerebrospinal fluid (CSF) inflammation is frequently seen in patients with aneurysmal subarachnoid hemorrhage (aSAH) requiring an external ventricular drain. Its role as a physiological response to the extravasation of blood, as a driver of delayed cerebral ischemia (DCI) and chronic hydrocephalus, or as a marker of ventriculostomy-related infection remains controversial. This study explored the associations between longitudinal routine CSF parameters and the development of DCI, shunt-dependent hydrocephalus, and functional neurological outcome in a large aSAH cohort.

**Methods:**

In this retrospective study, 702 samples from 169 patients with aSAH with at least three serial ventricular CSF samples from an external ventricular drain within the first 14 days after aSAH were included. Routine CSF parameters—that is, leukocyte count (CC) and erythrocyte count (EC), CSF lactate (LCT), and CSF/plasma glucose ratio—were considered. For bivariate analysis, values and time points of peak CC, EC, LCT, and trough CSF/plasma glucose ratio, respectively, and mean values in the early, middle, and late phase after aSAH were considered. For the analysis of longitudinal CSF parameter dynamics, generalized linear mixed-effects modeling was applied. Patients were compared according to the development of DCI, shunt-dependent hydrocephalus, and dichotomized short-term and long-term functional neurological outcome.

**Results:**

Longitudinal CC followed a nonlinear course over the first 14 days, with a peak around day 4 to day 8 after aSAH with a temporal relationship between peak CC and the occurrence of DCI. Higher initial EC with a more rapid decline was associated with favorable functional outcome, whereas persistently elevated LCT levels were seen in patients with unfavorable outcome. Patients with microbiologically confirmed ventriculostomy-related infection did not stand out significantly regarding CC absolute values or longitudinal dynamics.

**Conclusions:**

Intrathecal inflammation during the first 14 days after aSAH with a peak around day 7 seems to be a physiological response to the extravasation of blood into the CSF space. In patients with favorable outcome, EC decay was more rapid, indicating a more efficient clearance of blood from the subarachnoid space.

**Supplementary Information:**

The online version contains supplementary material available at 10.1007/s12028-025-02406-x.

## Introduction

Aneurysmal subarachnoid hemorrhage (aSAH) remains a severe life-threatening neurological disease associated with high morbidity and mortality [1, 2]. In patients surviving the immediate effects of aSAH, high morbidity is largely caused by secondary brain damage due to delayed cerebral ischemia (DCI). The pathophysiology of DCI is multifactorial and remains incompletely understood [3]. The release of hemoglobin from erythrolysis into the subarachnoid space and its breakdown products in the cerebrospinal fluid (CSF) trigger a complex cascade, leading to DCI [4–6]. In patients with acute hydrocephalus, an external ventricular drain (EVD) is inserted to reestablish CSF diversion and to monitor intracranial pressure. Additionally, continuous access to the intracranial CSF space by means of an EVD offers a window to monitor the intrathecal inflammatory response after aSAH but also poses the risk of ventriculostomy-related infections (VRIs) [7, 8]. The differentiation between an inflammatory response as part of the physiological degradation of blood constituents or one caused by an intrathecal infection represents a conundrum in clinical practice. Furthermore, inflammation may underlie DCI and be associated with the development of chronic hydrocephalus, requiring permanent CSF diversion by means of ventriculoperitoneal shunt placement [9–11]. Indeed, conflicting results have been reported regarding the association of routine ventricular CSF parameters and the occurrence of aSAH complications and neurological outcome [12].

In this explorative retrospective study, we comprehensively assess the association of longitudinal routine CSF parameters with complications, such as DCI and shunt-dependent hydrocephalus, and outcome in aSAH in one of the largest cohorts reported thus far. As excessive blood contamination of CSF, as typically seen in aSAH, distorts the analysis of CSF protein contents, we focused on the routine CSF parameters of leukocyte count (CC), erythrocyte count (EC), CSF lactate (LCT), and CSF/plasma glucose ratio (GluQ).

## Materials and Methods

This retrospective study included only patients who gave written informed consent themselves or through their legal guardians for retrospective data collection. The study was approved by the local ethics committee (Kantonale Ethikkomission Zürich, BASEC-Nr. 2021-00631) and was conducted in accordance with the ethical standards laid down in the 2023 Declaration of Helsinki for research involving human study participants.

### Patients

**Fig. 1:**
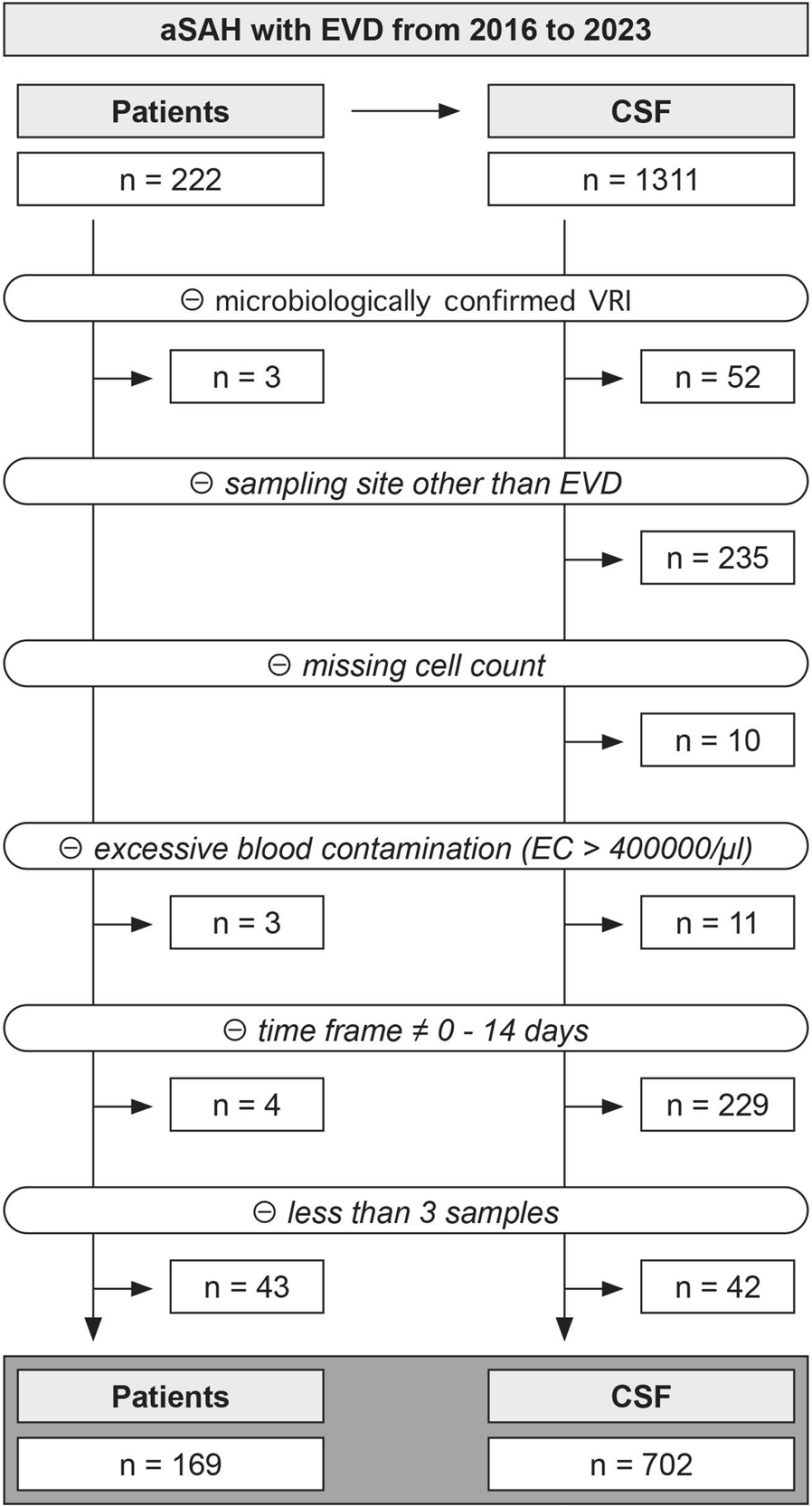
Patient and sample collection. Only ventricular samples from patients without VRI drawn from the EVD between day 0 and day 14 after aSAH were considered. Ultimately, only patients with at least three remaining serial samples were included in the analysis. aSAH, aneurysmal subarachnoid hemorrhage; CSF, cerebrospinal fluid; EC, erythrocyte count; EVD, external ventricular drain; VRI, ventriculostomy-related infection

Consecutive patients with aSAH and without microbiologically proven VRI treated in the neurocritical care unit of the University Hospital Zurich, Switzerland, between January 2016 and December 2023 were included. CSF samples were included for analysis if they (1) were drawn from an EVD, (2) had EC and LC available, (3) were without excessive blood contamination defined by an EC of < 400,000/μL, (4) were drawn between day 0 and day 14 after subarachnoid hemorrhage (SAH), and (5) were drawn from patients with at least three serial samples. Details on the number of excluded samples and of the remaining patients/samples thereafter are outlined in Fig. 1.

### Data Collection

All clinical data were retrospectively collected from the hospital’s electronic health records. CSF analyses were retrieved from the database of the Cerebrospinal Fluid Laboratory of the Department of Neurology, University Hospital Zurich, Switzerland.

### Definitions

Severity of aSAH was assessed by the World Federation of Neurosurgical Societies classification [13], the Hunt and Hess classification [14], and the Fisher scale [15]. World Federation of Neurosurgical Societies grades 4 and 5 were considered poor-grade aSAH. DCI was defined as a new perfusion deficit and/or infarction on computed tomography not attributable to other causes, such as endovascular treatment or clipping, and not already present within 48 h after aneurysm treatment [16].

Follow-up for functional neurological outcome at 1 to 3 months (short term) and 6 to 12 months (long term) was assessed retrospectively based on available electronic health records by neurocritical care physicians using the Glasgow Outcome Scale-Extended (GOSE) and was dichotomized into favorable (GOSE > 5) and unfavorable outcome (GOSE ≤ 4) [17, 18].

VRI was defined based on the recommendations of the Infectious Disease Society of America [19]. At our institution, eubacterial polymerase chain reaction (ePCR) is used in addition to CSF culture to confirm or refute a diagnosis of VRI. Hence, VRI was suspected (and treated with antibiotics) whenever at least two of the following criteria were present: (1) clinical signs of VRI, such as tympanic/bladder temperature of ≥ 38.3 °C or brain temperature > 38 °C measured within the brain tissue, meningeal irritation, and/or unclear neurological deterioration not better explained by an alternative cause (e.g., increasing intracranial pressure, progression of hemorrhage, or cerebral vasospasm); (2) CSF cell count > 500/μL (set as a local cut-off both based on institutional experience and previous research [20]); and (3) elevated markers of systemic inflammation (i.e., C-reactive protein > 5 mg/L, procalcitonin [PCT] > 0.1 μg/L, and/or white blood cell count > 9.6G/L not better accounted for by an alternative cause). VRI was only considered confirmed when CSF cultures and/or CSF ePCR were positive in cases of suspected VRI.

### EVD Management

Institutional EVD management comprises the neurosurgical placement of an EVD under sterile conditions in case of hydrocephalus/ventriculomegaly and the administration of a preoperative prophylactic intravenous single shot of cefuroxime. Silver impregnated lines (SilverlineR, Spiegelberg, Germany) are used. Regular CSF sampling for VRI screening is performed under sterile conditions twice per week and whenever clinically indicated. After discarding the first 2 mL of CSF, 3 mL are withdrawn from the EVD and sent in for laboratory investigations.

### CSF Analyses

CSF CC and EC were obtained by manual cell counting in a Fuchs-Rosenthal chamber and are given in cells/μL. Whenever necessary, CC was corrected for the presence of erythrocytes, assuming one leukocyte per 1000 to 2000 erythrocytes. The mean of the resulting range was used for statistical purposes, indicated by the following formula: CC_corrected_ = (CC_measured_ − [1.5 × EC])/1000. The internationally accepted reference range for ventricular CC is < 2 cells/μL, and any value above this was regarded as a surrogate of cellular inflammation irrespective of its cause. Crude cell count differentials were graded as predominantly mononuclear (> 66% mononuclear), mixed mononuclear and polynuclear, and predominantly polynuclear (> 66% polynuclear). LCT and GluQ were measured enzymatically.

To calculate mean values of CSF parameters of different disease phases, the first 2 weeks after aSAH were divided into an early (days 0–4), a middle (days 5–9), and a late phase (days 10–14).

To obtain mean crude cell count differentials, the categories predominantly mononuclear, mixed mononuclear and polynuclear, and predominantly polynuclear were transformed into 0, 1, and 2 before mean calculation, and mean values were transformed back into categorical form after mathematical rounding (i.e., a value of 0.5 would be rounded to 1 and transformed back into mixed mononuclear/polynuclear).

### Statistics

For descriptive statistics, continuous variables are given as median (minimum–maximum) and categorical variables as absolute number (%).

For correlation analyses between aSAH extent (Fisher classification) and EC values, Spearman’s rank correlation was used, assuming nonnormally distributed data.

Neurological short-term and long-term outcome and the occurrence of DCI, VRI, and shunt-dependent hydrocephalus were considered dichotomized response/grouping variables. Bivariate between-group analyses were performed using the Mann–Whitney *U*-test or Kruskal–Wallis test for continuous variables as appropriate and the *χ*^2^ test for categorical variables. For repeated-measures analyses, the Friedman test was used.

To illustrate the discriminative performance of peak and mean CSF parameter variables, receiver operating characteristic curves were employed.

To account for varying individual sample numbers and days, generalized linear mixed-effects models (GLMEMs) were applied to CSF parameters as a function of time, assuming fixed effects of time and—if indicated—grouping variable (i.e., complication or outcome), including an interaction term between the two. To account for nonlinear dynamics, a third-degree polynomial GLMEM with random intercepts and slopes was applied initially (Wilkinson notation: Response ~ Group*Time^3^ + [1 + Time|ID]). Statistically insignificant terms were then subtracted iteratively to find the best-fitting model based on the Bayesian information criterion, ultimately yielding a first-degree GLMEM with random intercepts for EC, LCT, and GluQ (Wilkinson notation: Response ~ Group*Time + [1|ID]) and a third-degree polynomial GLMEM with random intercepts and slopes for CC, respectively (Wilkinson notation: Response ~ Group*Time^3^ + [1 + Time|ID]). To visualize the results of the GLMEM on a group basis, their predicted response was plotted, assuming no individual random effects.

All statistical analyses and their graphical illustrations were performed using MATLAB R2022b (The MathWorks Inc., Natick, MA). Statistical significance was accepted at *P* < 0.05.

Incomplete patient information, including loss to follow-up, was treated as missing data and is provided as percentage when appropriate.

## Results

A total of 702 CSF samples drawn between day 0 and day 14 from 169 patients were available for analysis. Day- and phase-wise data availability across the studied time frame for each CSF parameter studied can be found in Supplementary Fig. 1. Of all 169 patients, 107 were female (63.3%), with a median age of 58.6 years (28.7–84.5). The majority of patients suffered from poor-grade SAH (63.3%). Detailed patient characteristics can be found in Table 1.

**Table 1:** Patient characteristics

Age	58.6 (28.7–84.5)
*Sex*	
Female	107 (63.3%)
*CSF samples*	
Number of CSF samples per patient	4 (3–7)
Patients with samples in the early phase	151 (83.3%)
Patients with samples in the mid phase	162 (95.9%)
Patients with samples in the late phase	134 (79.3%)
Day of first CSF sample	2 (0–9)
Day of last CSF sample	12 (5–14)
**ICU length of stay (days)**	**20 (4**–**104)**
*WFNS classification*	
WFNS 1	18 (10.7%)
WFNS 2	34 (20.2%)
WFNS 3	9 (5.4%)
WFNS 4	55 (32.7%)
WFNS 5	52 (31%)
n.a	1 (0.6%)
*Hunt and Hess classification*	
HH 1	16 (9.6%)
HH 2	22 (13.2%)
HH 3	42 (25.1%)
HH 4	45 (26.9%)
HH 5	42 (25.1%)
n.a	2 (1.2%)
*Fisher classification*	
Fisher 1	2 (1.2%)
Fisher 2	1 (0.6%)
Fisher 3	48 (28.6%)
Fisher 4	117 (69.6%)
n.a	1 (0.6%)
*Treatment of aneurysm*	
Clipping	52 (30.8%)
Coiling	107 (63.3%)
Other	9 (5.3%)
Conservative	1 (0.5%)
**Delayed cerebral ischemia**	**68 (40.2%)**
Perfusion deficit only	26 (15.4%)
Infarction	42 (24.9%)
Intraarterial nimodipine and/or ballon angioplasty	46 (27.2%)
**Death on ICU**	**14 (7.7%)**
*Outcome at 1–3 months follow-up*	
Favorable	40 (21.9%)
Unfavorable	139 (76%)
n.a	4 (2.2%)
*Outcome at 6–12 months follow-up*	
Favorable	60 (32.8%)
Unfavorable	92 (50.3%)
n.a	31 (16.9%)
*Shunt*	
Shunt-dependency	110 (60.1%)
Day of shunt placement	26 (14–215)

CSF, cerebrospinal fluid; GOSE, Glasgow Outcome Scale-Extended; ICU, intensive care unit; n.a., not available; WFNS, World Federation of Neurosurgical Societies. Continuous variables are given in median (minimum–maximum) and categorical variables are given in count (percentage)

### CSF CC and Differential Follow a Nonlinear Time Course with a Peak on Days 4 to 8 After SAH

**Fig. 2:**
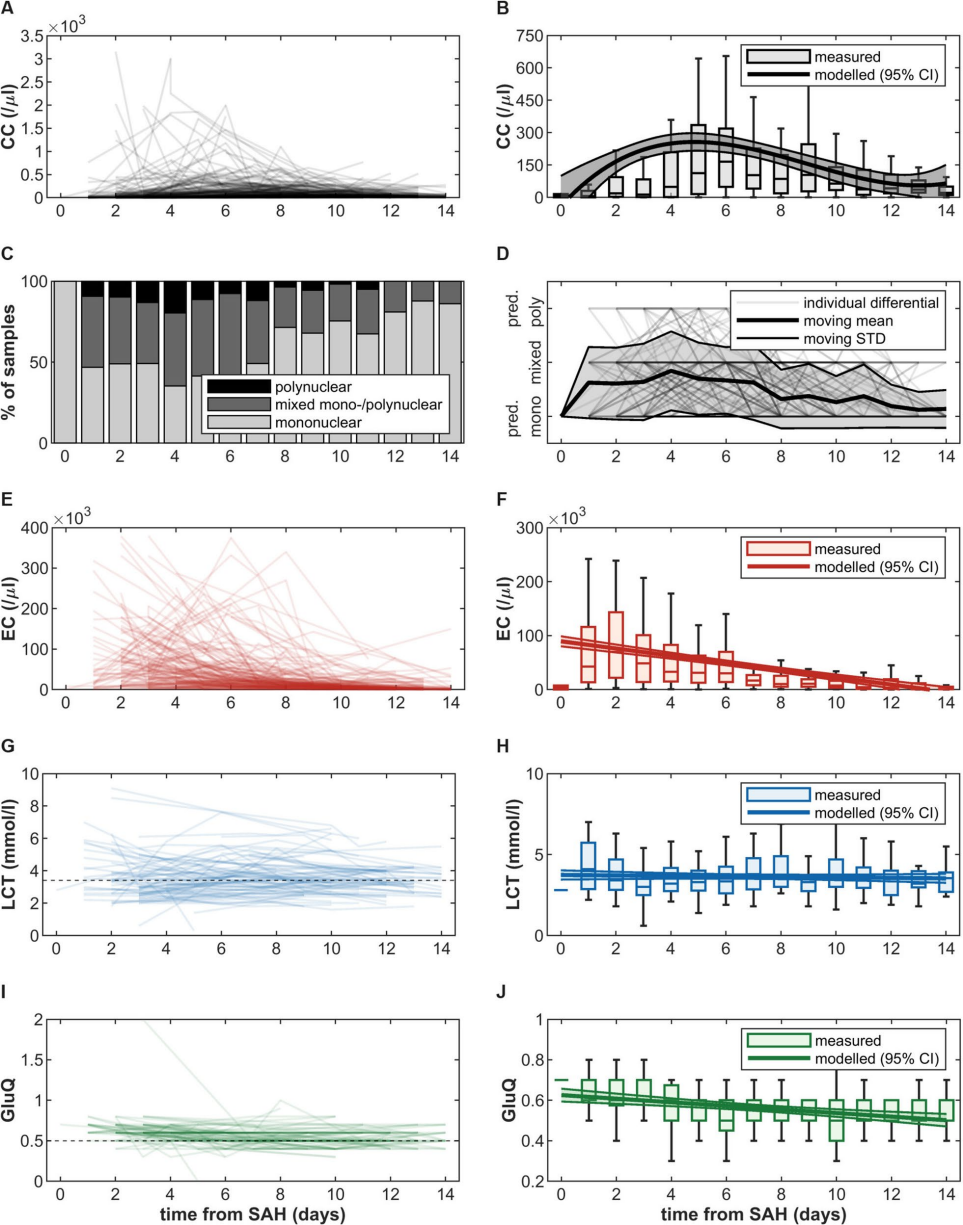
Longitudinal dynamics of routine cerebrospinal fluid (CSF) parameters. **A** Spaghetti plot of individual cell count (CC) courses. **B** Generalized linear mixed-effects model (GLMEM) of longitudinal CC course and day-wise distribution as traditional boxplots. **C** Distribution of crude cell count differentials categorized as *predominantly mononuclear*, *mixed mono-/polynuclear* and *predominantly polynuclear*. **D** Moving median of ordinalized crude differential. **E** Spaghetti plot of individual erythrocyte count (EC) courses. **F** GLMEM of longitudinal EC course and day-wise distribution as traditional boxplots. **G** Spaghetti plot of individual CSF lactate (LCT) courses. Dashed line: upper limit of normal of 3.4 mmol/l. **H** GLMEM of longitudinal LCT course and day-wise distribution as traditional boxplots. **I** Spaghetti plot of individual CSF/plasma glucose ratio (GluQ) courses. Dashed line: lower limit of normal of 0.5. **J** GLMEM of longitudinal GluQ course and day-wise distribution as traditional boxplots. CC, cell count; CI, confidence interval; CSF, cerebrospinal fluid; EC, erythrocyte count; GLMEM, generalized linear mixed-effects model; GluQ, CSF/plasma glucose ratio; LCT, lactate; SAH, subarachnoid hemorrhage; STD, standard deviation

CC increased in the first days after aSAH and then gradually decreased (Fig. 2a, b). Median peak CC was 210.5/μL (8–3158/μL) and occurred on day 7 (days 1–14). The day of peak CC was distributed relatively normally, with 56.8% of patients displaying their peak on days 4 to 8. Similarly, this was also the time with the highest proportion of mixed mononuclear/polynuclear and predominantly polynuclear crude differentials (Fig. 2c, d).

**Fig. 3:**
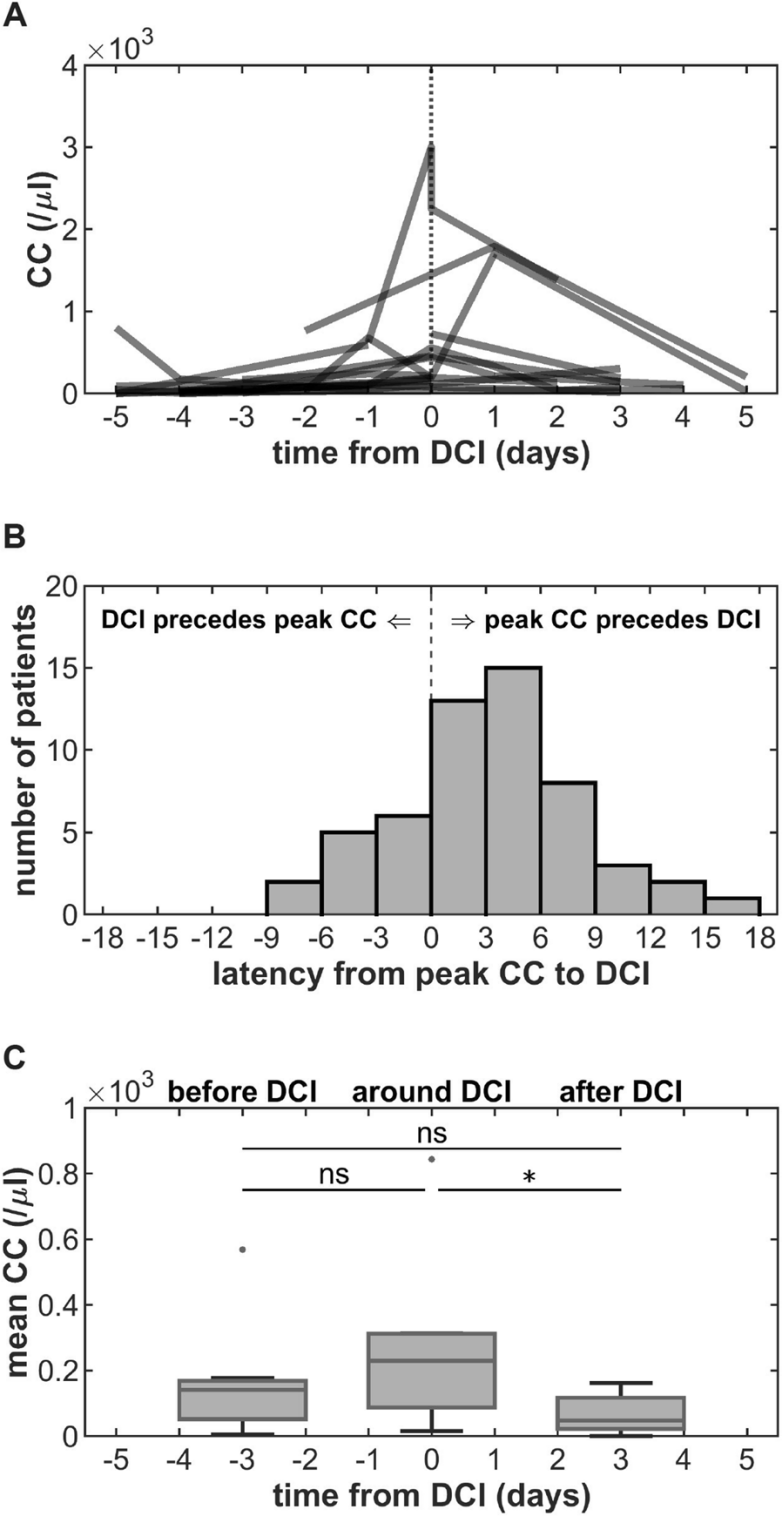
Associations of cerebrospinal fluid (CSF) peak cell count (CC) and the occurrence of delayed cerebral ischemia (DCI). **A** Longitudinal CC centered around the occurrence of DCI. **B** Latency from peak CC to DCI. **C** Comparison of mean CC before (four to two days before DCI), around (one day before to one day after DCI), and after DCI (two to four days after DCI) in a subgroup of patients (*n* = 9). Statistical significance: **p < 0.001; *p < 0.05; ns, not significant; CC, leukocyte count; DCI, delayed cerebral ischemia

Patients with or without DCI or shunt-dependent hydrocephalus, as well as with unfavorable or favorable short-term and long-term functional outcomes, respectively, did not differ in their mean or peak CC values or longitudinal CC time course (Supplementary Figs. 2–5). However, there was a temporal relationship between peak CC and the occurrence of DCI, with peak CC frequently coinciding with or shortly preceding DCI (Fig. 3a, b). Median latency from peak CC to DCI was 3 days (− 8 to 16 days). In a small subgroup of patients (*n* = 9) with CSF samples available in the time frames of four to two days before DCI, one day before to one day after DCI, and two to four days after DCI, mean CC after DCI was significantly lower than mean CC around DCI (Fig. 3c).

### High Initial CSF EC and Rapid Decline Associate with Good Functional Outcome

Median EC in the first available sample was 47,000/μL and gradually decreased over time, although in 42.6% of patients, peak EC was reached later than in the first available sample (median peak EC 76,000/μL [270–380,000/μL], median days of peak EC 2 [0–9 days]). Importantly, EC in the first available sample did not correlate with SAH extent as assessed by the Fisher classification, but peak EC weakly did (Pearson’s *ρ* = 0.186, *P* = 0.016). However, the vast majority of patients were classified as Fisher 3 and 4 (Table 1).

**Fig. 4:**
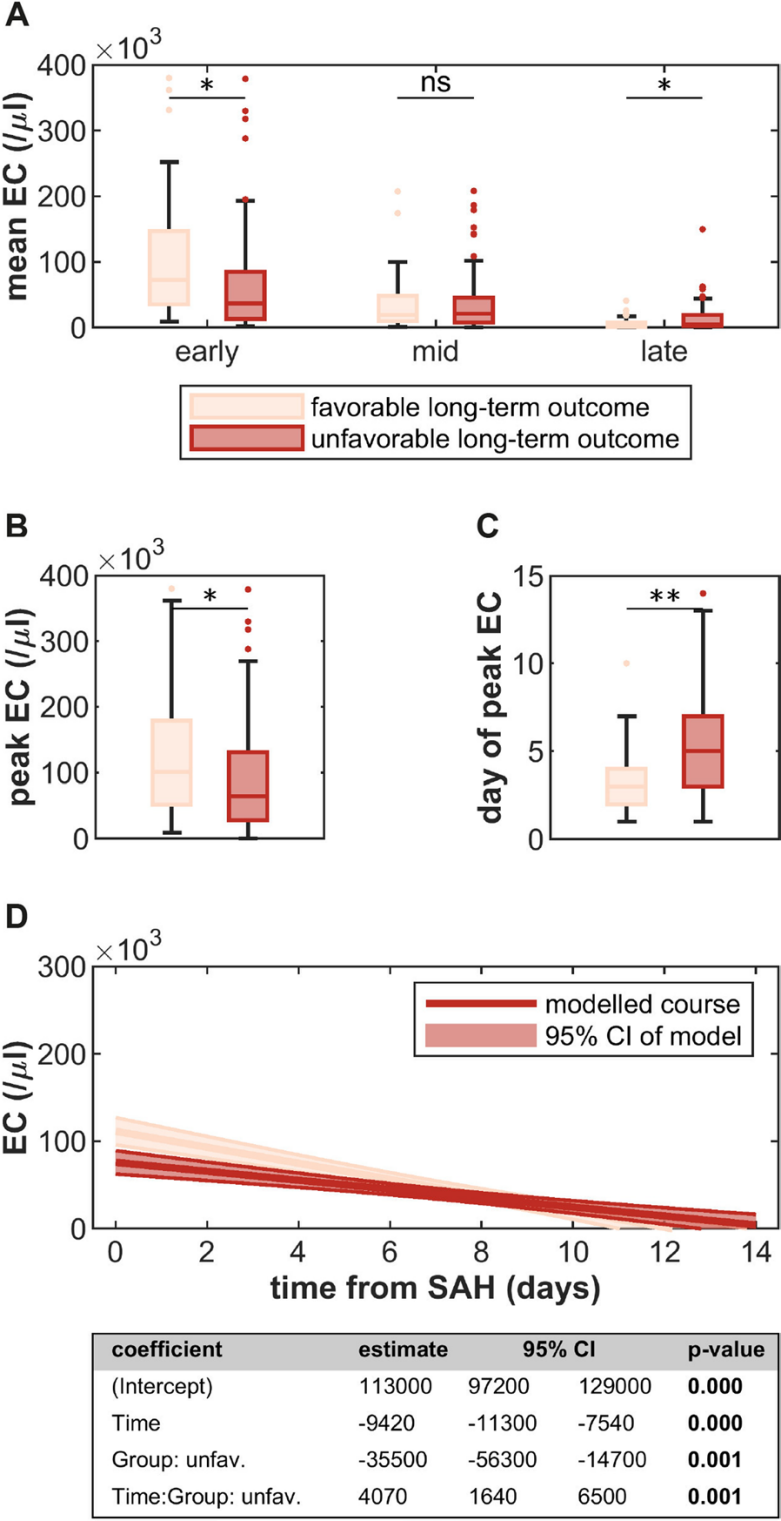
Associations of cerebrospinal fluid (CSF) erythrocyte counts (EC) and long-term functional outcome. **A** Comparison of early, mid and late mean EC between patients with favorable and unfavorable outcome. **B**, **C** Comparison of peak EC and day of peak EC between patients with favorable and unfavorable outcome. **D** Generalized linear mixed-effects model (GLMEM) of EC decay over time in patients with favorable and unfavorable outcome. Statistical significance: **p < 0.001; *p < 0.05; ns, not significant. EC, erythrocyte count; SAH, subarachnoid hemorrhage

Patients with favorable long-term functional outcome had higher early mean EC and peak EC values, reached their peak EC earlier, and had lower late mean EC values than patients with unfavorable long-term functional outcome (Fig. 4a–c). Consistently, GLMEM revealed a significantly higher intercept and a steeper slope of EC decay in those with favorable outcome (Fig. 4d). A similar, albeit less pronounced, effect was seen for short-term functional outcome and shunt-dependent hydrocephalus but not for DCI (Supplementary Figs. 6–8).

### LCT is Consistently Elevated in Patients with Unfavorable Functional Outcome

**Fig. 5:**
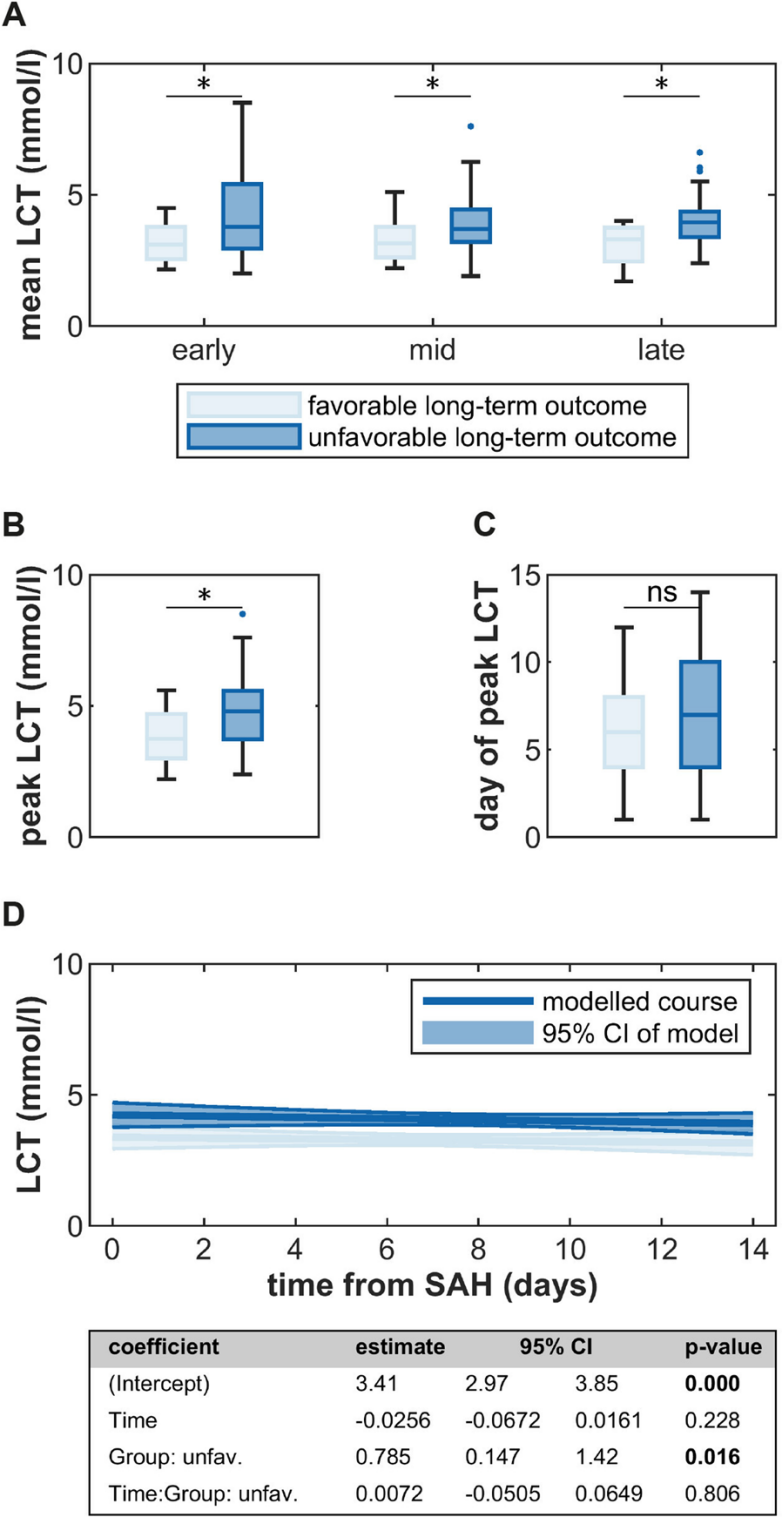
Associations of cerebrospinal fluid (CSF) lactate (LCT) and long-term functional outcome. **A** Comparison of early, mid and late mean LCT between patients with favorable and unfavorable outcome. **B**, **C** Comparison of peak LCT and day of peak LCT between patients with favorable and unfavorable outcome. **D** Generalized linear mixed-effects model (GLMEM) of EC decay over time in patients with favorable and unfavorable outcome. Statistical significance: **p < 0.001; *p < 0.05; ns, not significant. LCT, cerebrospinal fluid lactate; SAH, subarachnoid hemorrhage

**Fig. 6:**
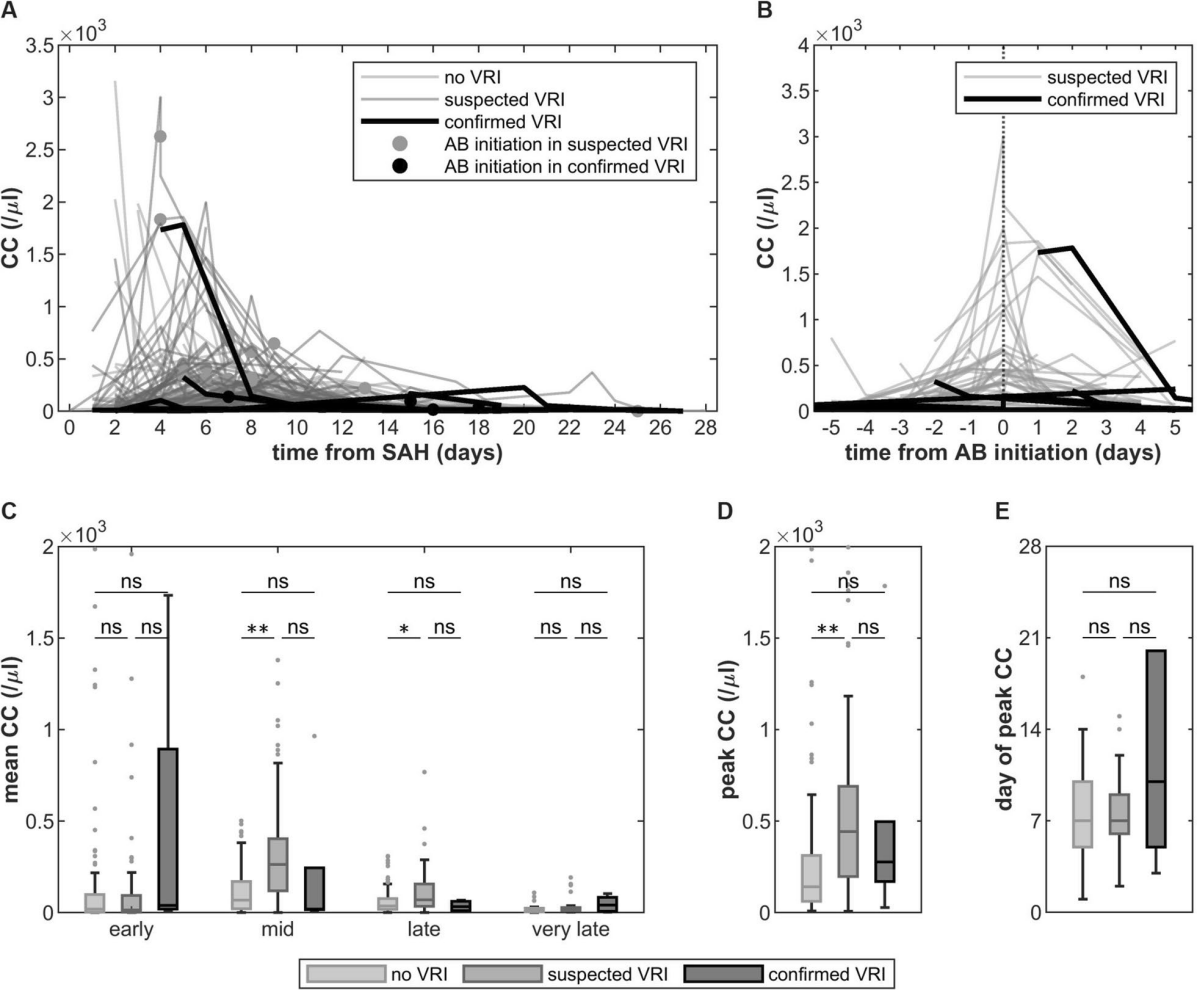
Longitudinal cell count courses from day 0 to day 28 after aneurysmal subarachnoid hemorrhage (aSAH) in patients without, with suspected and with confirmed ventriculostomy-related infection (VRI). **A** Spaghetti plot of individual cell count courses and timepoint of antibiotics initiation. **B** Longitudinal cell count centered around the day of antibiotics initiation. **C** Mean cell count in the early (days 0–4), mid (days 5–9), late (days 10–day 14) and very late phase (days 15–28) after aSAH. **D** Peak cell count. **D** Day of peak cell count. AB, antibiotics; CC, cell count; SAH, subarachnoid hemorrhage; VRI, ventriculostomy-related infection

LCT showed only minimal change over time but was pathologically elevated in 47.7% of samples (i.e., > 3.4 mmol/L) (Fig. 2g, h). Patients with unfavorable outcome had significantly higher mean early, middle, and late as well as peak LCT values than those with favorable outcome (Fig. 5a–c). This was seen across short-term and long-term functional outcome and was replicated by GLMEM, suggesting an intercept difference of 0.767 mmol/L and 0.785 mmol/L for short-term and long-term functional outcome, respectively (Supplementary Fig. 11 and Fig. 6d). No differences in LCT levels were seen regarding the development of DCI or shunt-dependent hydrocephalus (Supplementary Figs. 9–11). Similarly, GluQ declined slowly over time but remained normal (i.e., > 0.5) in most patients and did not associate with complication or outcome variables (Supplementary Figs. 12–15).

### CSF Cell Count Fails to Identify VRI

To assess the effect of VRI on CC, samples from three patients with aSAH and confirmed VRI who were initially excluded from the study (Fig. 1) and an additional three patients with aSAH and confirmed VRI from 2013 to 2016 were compared against the aSAH cohort. As 3 of 6 VRI diagnoses were made after day 14 after aSAH, samples from day 0 to day 28 after aSAH were considered for the analysis. Patient details can be found in Supplementary Table 1.

The 28-day CC courses of patients with confirmed VRI did not visually stand out from patients without or with only suspected VRI (i.e., without positive CSF culture and/or ePCR) (Fig. 6a). Frequently, initiation of antibiotics occurred on the day of peak CC in those with suspected VRI but not in those with confirmed VRI (Fig. 6b). Notably, statistically significant differences in middle and late mean CC as well as peak CC were found between patients without VRI and patients with suspected VRI but did not concern those with confirmed VRI (Fig. 6c–e).

## Discussion

In this study, we explored the association of ventricular routine CSF parameters with functional neurological outcome and complications of aSAH, such as DCI, shunt-dependent hydrocephalus, and VRI, in a total of 702 CSF samples from 169 patients.

In concordance with previous studies [21], the cellular immune response to blood extravasation and erythrocytolysis in the subarachnoid space followed a nonlinear course, with a peak between day 4 and day 8 after aSAH, during which the cellular immune response was composed of mononuclear and polynuclear cells—that is, lymphocytes and granulocytes. On the other hand, EC was highest in the first days after aSAH and gradually declined.

Dichotomization of patients according to functional outcome, the development of DCI, or the development of shunt-dependent hydrocephalus, respectively, revealed largely overlapping absolute CC values in all time periods, and the CC dynamics presented similarly. Notably, in patients with DCI, there was a temporal association between its occurrence and peak CC. Although intrathecal inflammation may intuitively be regarded as a driver of complications of aSAH, such as DCI or chronic hydrocephalus, it may, in fact, promote the clearance of toxic blood degradation products that may play pivotal roles in the mechanisms underlying secondary brain damage [4]. The interplay of intrathecal inflammation and DCI warrants further research.

Peak EC was higher—but occurred earlier—in patients with favorable outcome than in patients with unfavorable outcome. Importantly, however, initial EC does not necessarily reflect the extent of aSAH but may be influenced by the presence or absence of an intraventricular tamponade that may sequester erythrocytes and lead to a false-low EC. On the other hand, mean EC in the late phase—during which much of blood clearance ought to have taken place already—was lower in those with favorable outcome. Similarly, GLMEM revealed a higher EC intercept but a steeper slope of EC decay in patients with favorable outcome, suggesting that effective blood clearance may be associated with improved outcome. Indeed, this concept has recently been adopted into clinical practice [22].

CSF LCT is a metabolite of anaerobic glycolysis and mostly serves as a marker of central nervous system infection. However, acute brain injury may cause LCT elevations, too [23, 24]. Currently, its role as a prognostic marker—potentially serving as a surrogate of the extent of brain damage—remains largely unexplored. In this study, patients with poor short-term and long-term functional outcome presented with consistently higher LCT values than those with favorable outcome. Moreover, among all absolute CSF parameter values explored, LCT showed the best discriminatory power between favorable and unfavorable outcome (Supplementary Fig. 16).

Microbiologically confirmed VRI was rare (i.e., 1.4% in the screened aSAH population) but suspected in roughly one third of patients. CC dynamics in patients with confirmed VRI did not significantly differ from patients without VRI. In contrast, highest mean CC in the early and middle phases as well as peak CC were seen in patients with suspected VRI but lacking a definite microbiological diagnosis. Although culture and PCR–negative VRI cannot entirely be excluded in these patients, previous studies suggest that cessation of antibiotic treatment after receiving a negative PCR is safe, likely because of a false positive diagnosis of VRI [7]. These findings suggest that the initiation of antibiotic therapy for suspected VRI requires careful evaluation.

### Limitations

Several limitations must be considered when interpreting the results of this study. First, because of its retrospective nature, data scarcity (i.e., different sample numbers and sampling time points per patient) must be considered. Preanalytical aspects of CSF sampling, such as delivery times from bedside to laboratory, could not be considered. Furthermore, the complexity of the polynomial GLMEM employed for the study of CC dynamics may be prone to overfitting. Lastly, the low number of patients with microbiologically confirmed VRI limits the generalizability of the presented results, and any interpretation of statistical results ought to be made cautiously.

## Conclusions

Intrathecal cellular inflammation during the first 14 days after aSAH with a peak around day 7 seems to be a physiological response to the extravasation of blood into the CSF space. Although its role in the development of secondary brain damage remains incompletely understood, the temporal association of peak CC and the occurrence of DCI suggests that a high index of suspicion concerning DCI is advisable in those with pronounced peak CC. The differentiation of sterile from infectious intrathecal inflammation in this critical patient population remains a clinical conundrum in need of further biomarker research. On the other hand, slow EC clearance and persistently high LCT associate with unfavorable outcome. The former supports the use of additional lumbar drains to accelerate blood clearance, as suggested by clinical trials [22, 25]. Ultimately, prospective CSF studies will advance our pathophysiological understanding of secondary brain damage associated with aSAH.

## Supplementary Information

Below is the link to the electronic supplementary material.

Supplementary file1 (DOCX 3080 kb)
