# Supplementary material for: Association of Serial Ventricular Routine Cerebrospinal Fluid Findings with Complications and Outcome in Aneurysmal Subarachnoid Hemorrhage: A Retrospective Observational Study
Source: Neurocrit Care. 2025 Nov 14;44(2):445–55. doi: 10.1007/s12028-025-02406-x (PMC13053543; doi:10.1007/s12028-025-02406-x)
Supplement: Supplementary file 1 — Supplementary file1 (DOCX 3080 kb) [file 12028_2025_2406_MOESM1_ESM.docx]

**Association of Serial Ventricular Routine Cerebrospinal Fluid Findings with Complications and Outcome in Aneurysmal Subarachnoid Hemorrhage: A Retrospective Observational Study**

**– SUPPLEMENTARY MATERIAL –**

Claudio Togni^1, 2^, Mina Pasqualini^2^, Ignazio de Trizio, MD^2^, Francesca Casagrande, MD^2^, Federica Stretti, MD^2^, Emanuela Keller, MD^2, 3^, Giovanna Brandi, MD^2^

**Author Affiliations:**

1. Department of Neurology, Clinical Neuroscience Center, University Hospital Zurich and University of Zurich, Zurich, Switzerland
2. Institute of Intensive Care Medicine, University Hospital Zurich, University of Zurich, Zurich, Switzerland
3. Department of Neurosurgery, Clinical Neuroscience Center, University Hospital Zurich and University of Zurich, Zurich, Switzerland

**
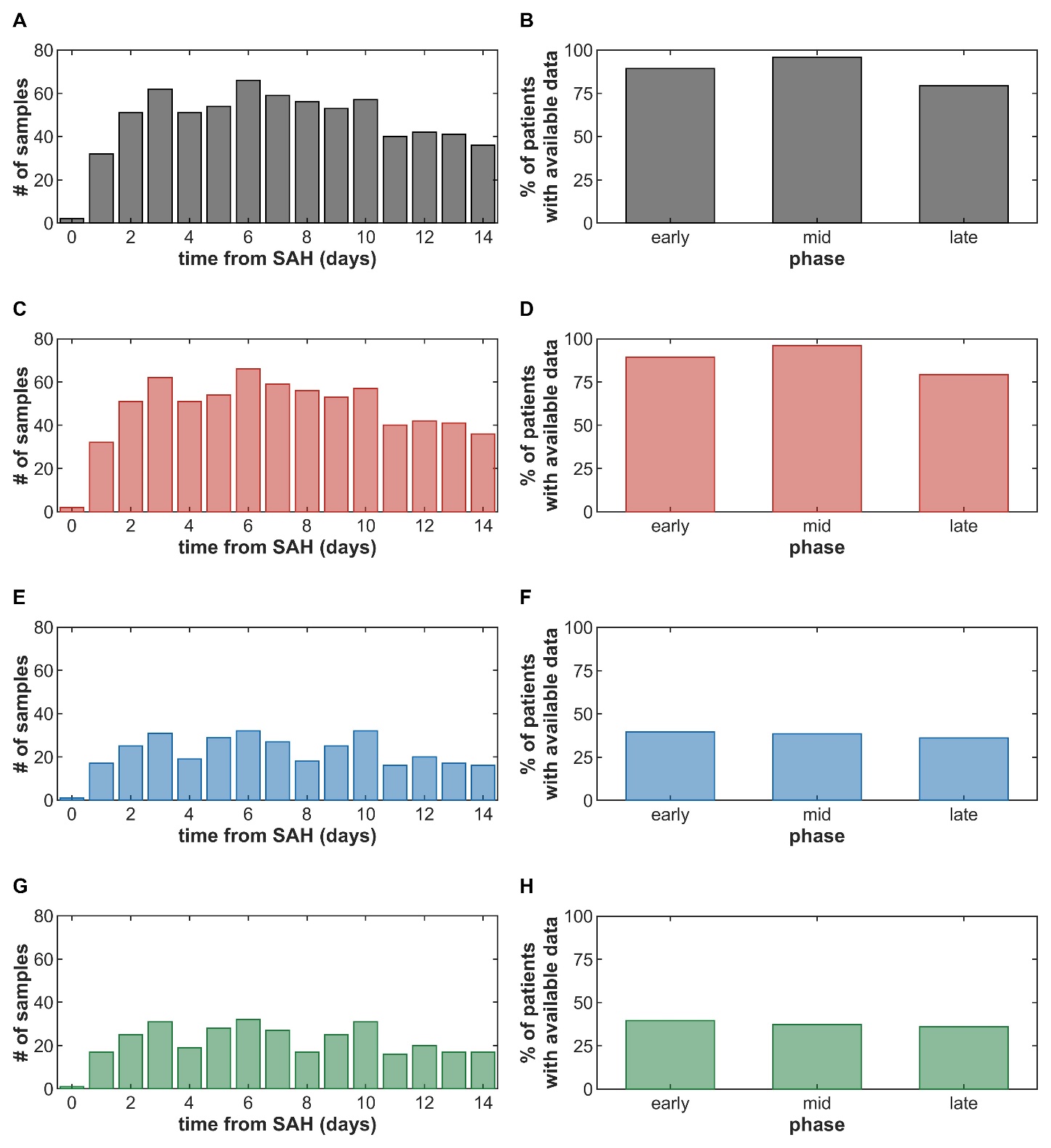
**

**Supplementary Figure 1:** Data Availability. Day- and phase-wise numbers of samples and the percentage of patients with available phase means for **(A, B)** cell count, **(C, D)** erythrocyte count, **(E, F)** lactate, and **(G, H)** glucose ratio, respectively.

**
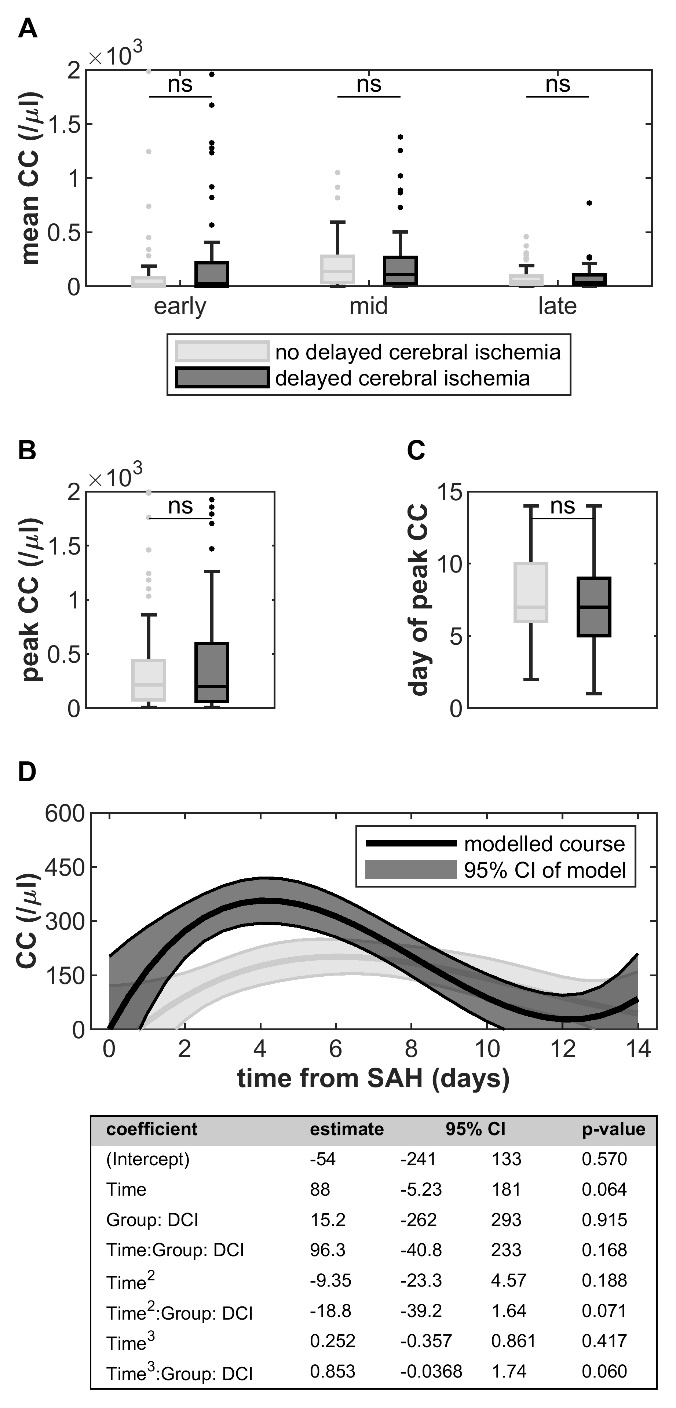
**

**Supplementary Figure 2:** Associations of cerebrospinal fluid (CSF) leukocyte counts (CC) and delayed cerebral ischemia (DCI). **(A)** Comparison of early, mid and late mean CC between patients without and with DCI. **(B, C)** Comparison of peak CC and day of peak CC between patients without and with DCI. **(D)** Generalized linear mixed-effects model (GLMEM) of CC over time in patients without and with DCI. Statistical significance: **, p < 0.001; *, p < 0.05; ns, not significant. CC, leukocyte count; DCI, delayed cerebral ischemia; SAH, subarachnoid hemorrhage.

**
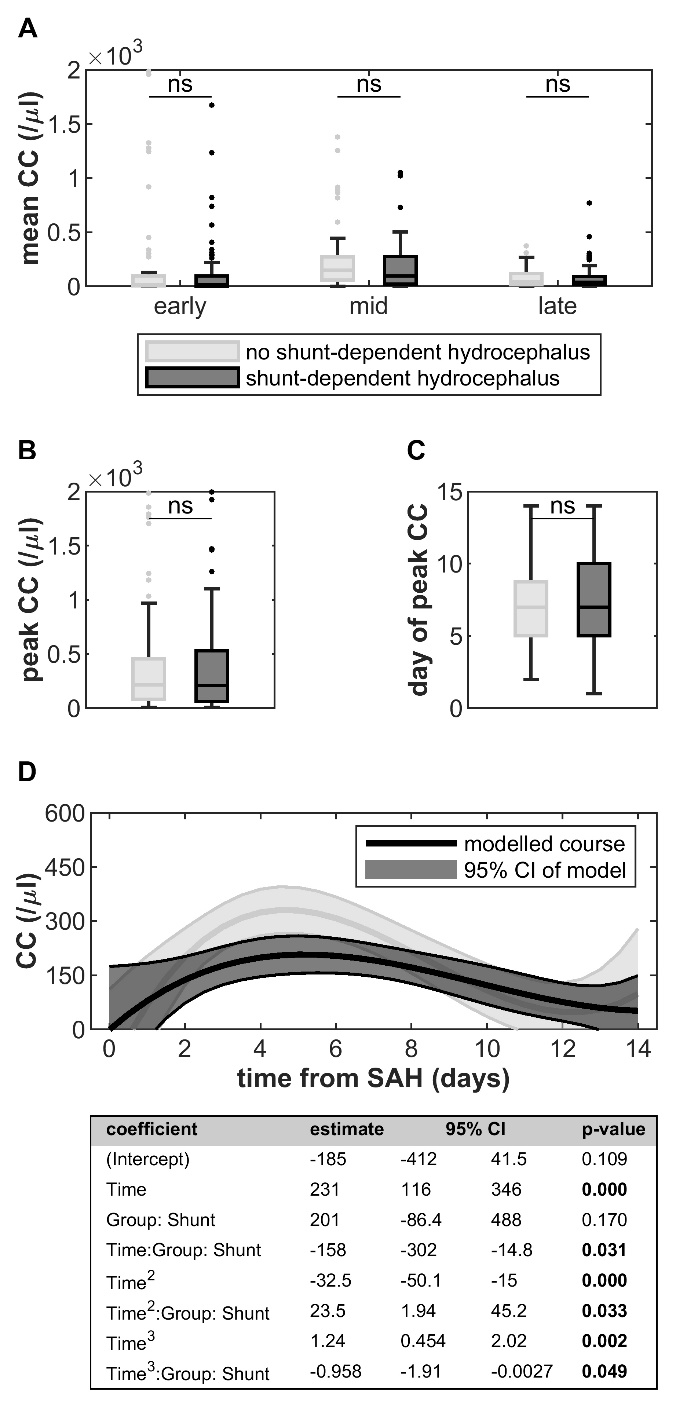
**

**Supplementary Figure 3:** Associations of cerebrospinal fluid (CSF) leukocyte counts (CC) and shunt-dependent hydrocephalus. **(A)** Comparison of early, mid and late mean CC between patients without and with shunt-dependent hydrocephalus. **(B, C)** Comparison of peak CC and day of peak CC between patients without and with shunt-dependent hydrocephalus. **(D)** Generalized linear mixed-effects model (GLMEM) of CC over time in patients without and with DCI. Statistical significance: **, p < 0.001; *, p < 0.05; ns, not significant. CC, leukocyte count; SAH, subarachnoid hemorrhage.

**
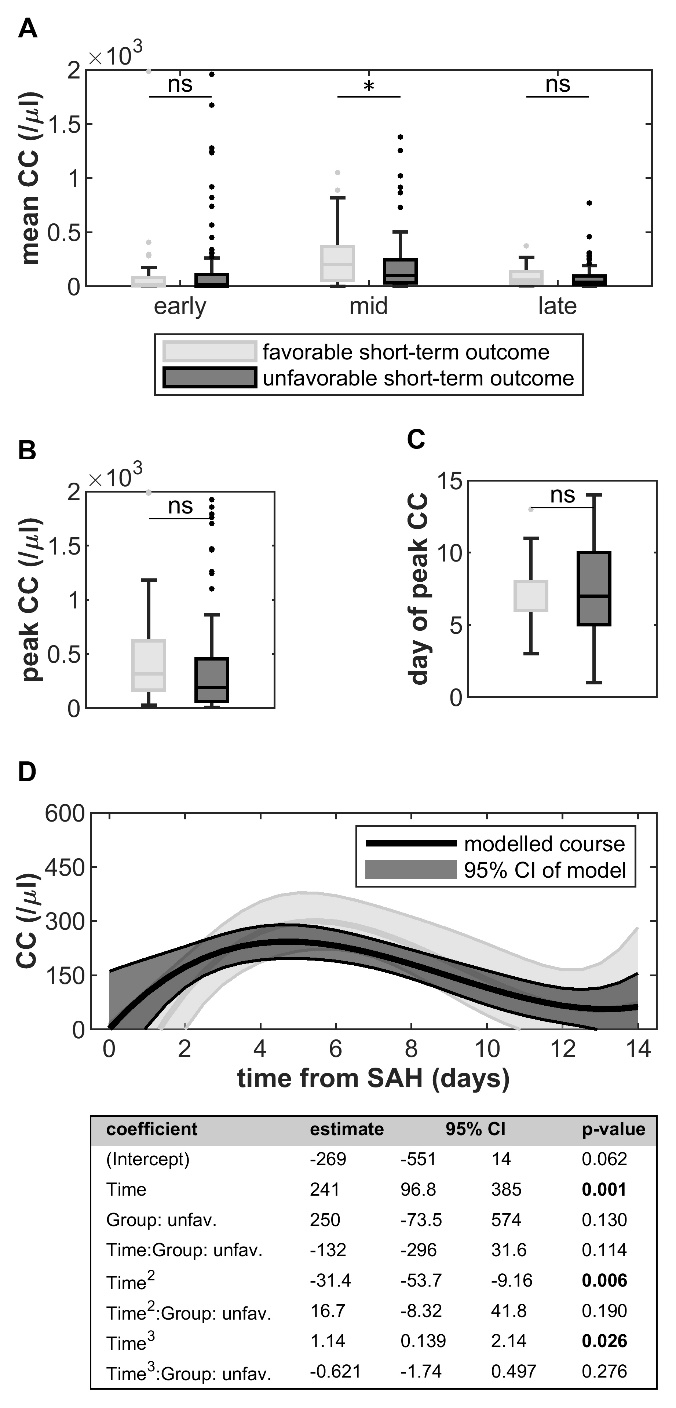
**

**Supplementary Figure 4:** Associations of cerebrospinal fluid (CSF) leukocyte counts (CC) and short-term functional outcome. **(A)** Comparison of early, mid and late mean CC between patients favorable and unfavorable outcome. **(B, C)** Comparison of peak CC and day of peak CC between patients favorable and unfavorable outcome. **(D)** Generalized linear mixed-effects model (GLMEM) of CC over time in patients with favorable and unfavorable outcome. Statistical significance: **, p < 0.001; *, p < 0.05; ns, not significant. CC, leukocyte count; SAH, subarachnoid hemorrhage.

**
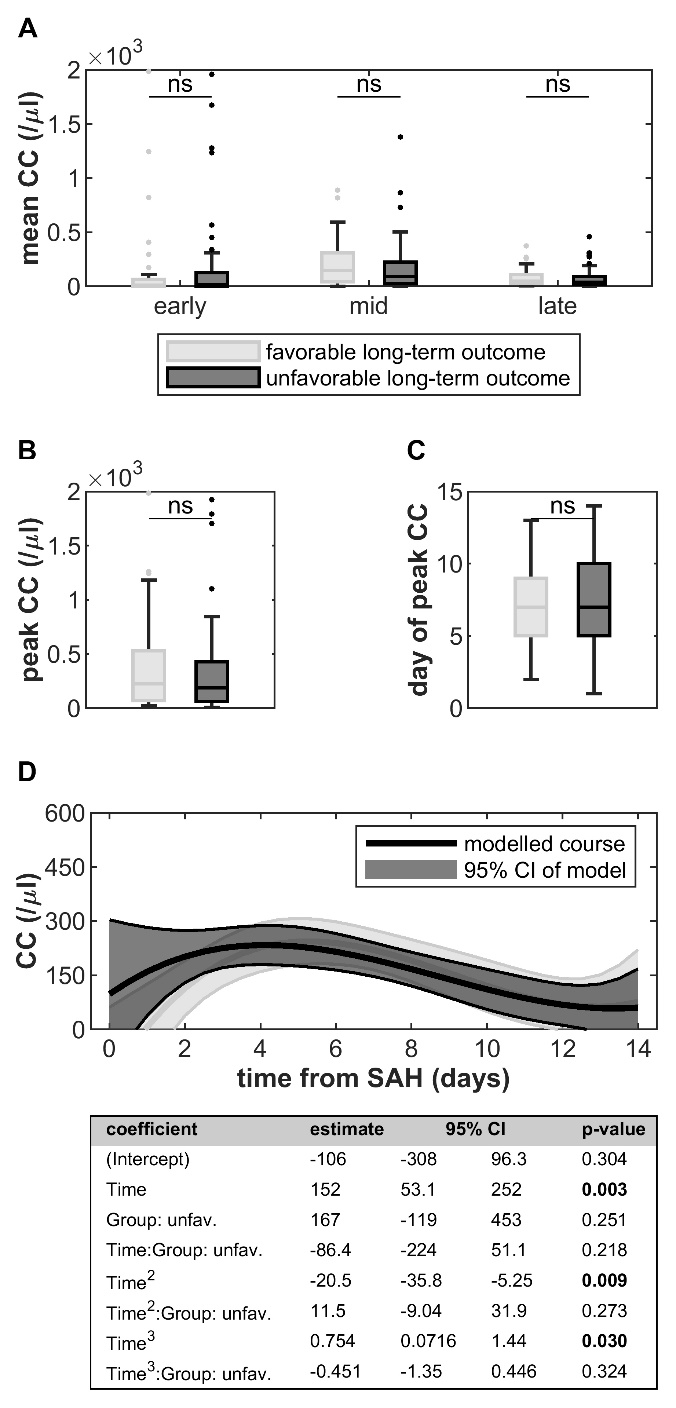
**

**Supplementary Figure 5:** Associations of cerebrospinal fluid (CSF) leukocyte counts (CC) and long-term functional outcome. **(A)** Comparison of early, mid and late mean CC between patients favorable and unfavorable outcome. **(B, C)** Comparison of peak CC and day of peak CC between patients favorable and unfavorable outcome. **(D)** Generalized linear mixed-effects model (GLMEM) of CC over time in patients with favorable and unfavorable outcome. Statistical significance: **, p < 0.001; *, p < 0.05; ns, not significant. CC, leukocyte count; SAH, subarachnoid hemorrhage.

**
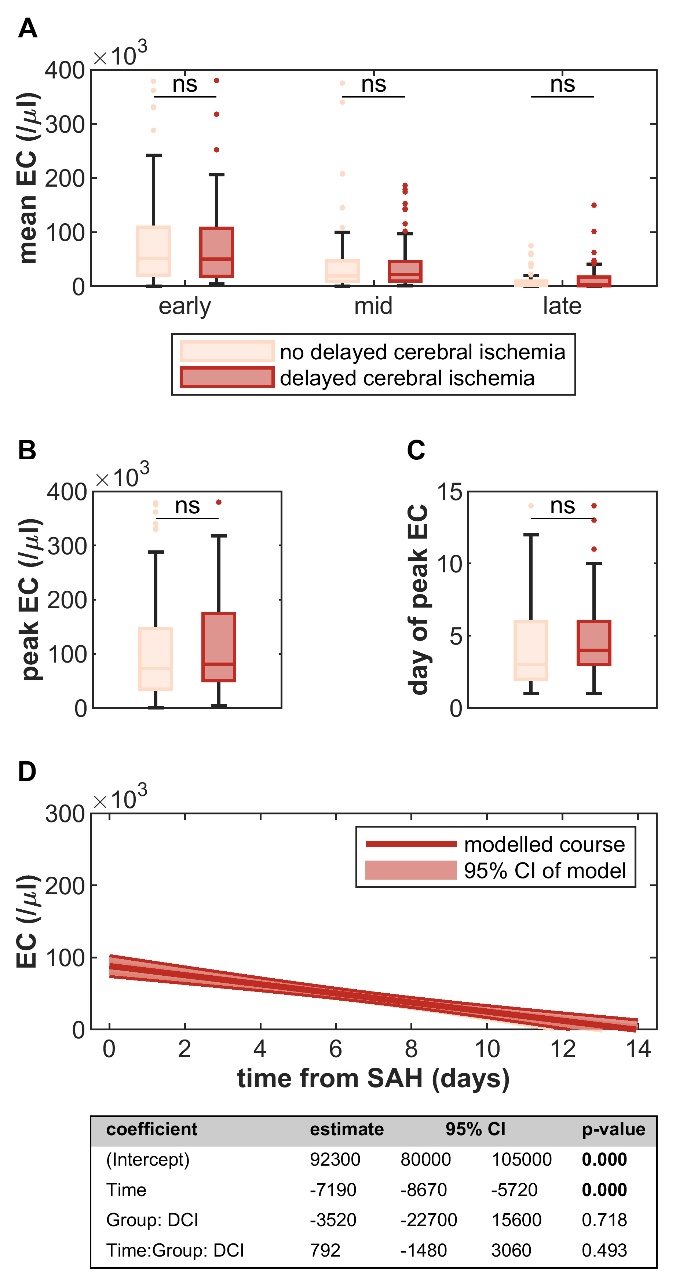
**

**Supplementary Figure 6:** Associations of cerebrospinal fluid (CSF) erythrocyte counts (EC) and delayed cerebral ischemia (DCI). **(A)** Comparison of early, mid and late mean EC between patients without and with DCI. **(B, C)** Comparison of peak EC and day of peak EC between patients without and with DCI. **(D)** Generalized linear mixed-effects model (GLMEM) of EC decay over time in patients without and with DCI. Statistical significance: **, p < 0.001; *, p < 0.05; ns, not significant. EC, erythrocyte count; DCI, delayed cerebral ischemia; SAH, subarachnoid hemorrhage.

**
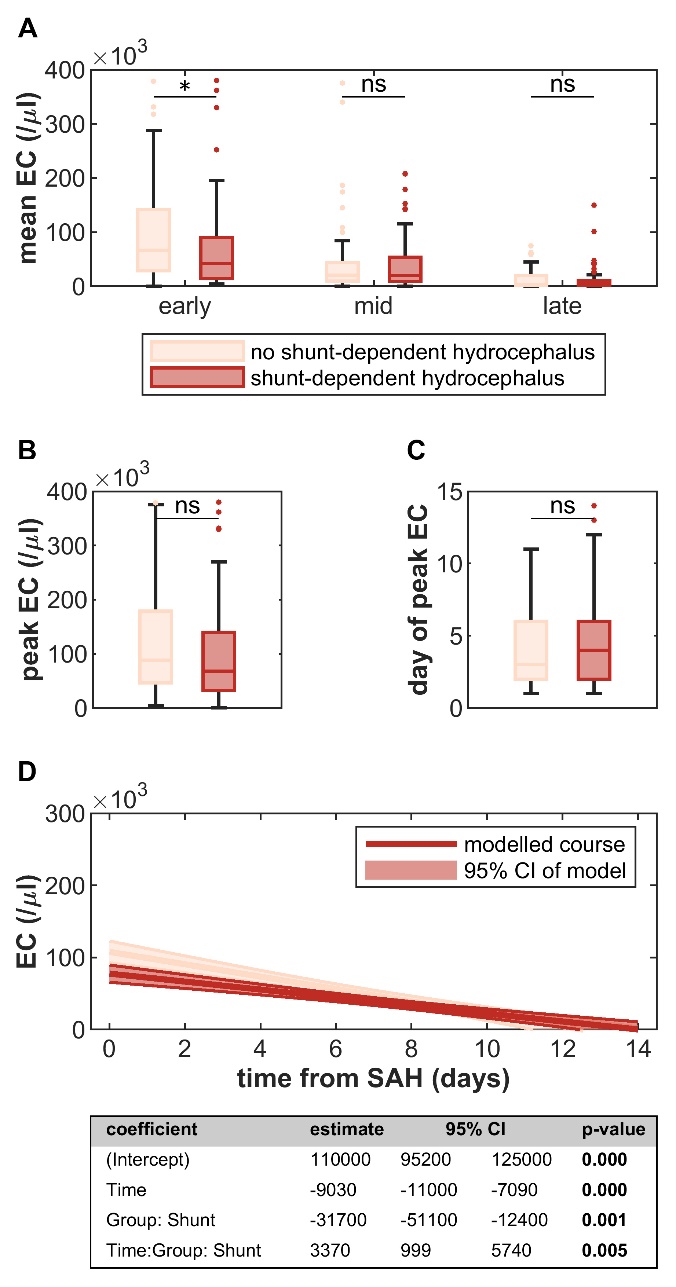
**

**Supplementary Figure 7:** Associations of cerebrospinal fluid (CSF) erythrocyte counts (EC) and shunt-dependent hydrocephalus. **(A)** Comparison of early, mid and late mean EC between patients without and with shunt-dependent hydrocephalus. **(B, C)** Comparison of peak EC and day of peak EC between patients without and with shunt-dependent hydrocephalus. **(D)** Generalized linear mixed-effects model (GLMEM) of EC decay over time in patients without and with shunt-dependent hydrocephalus. Statistical significance: **, p < 0.001; *, p < 0.05; ns, not significant. EC, erythrocyte count; SAH, subarachnoid hemorrhage.

**
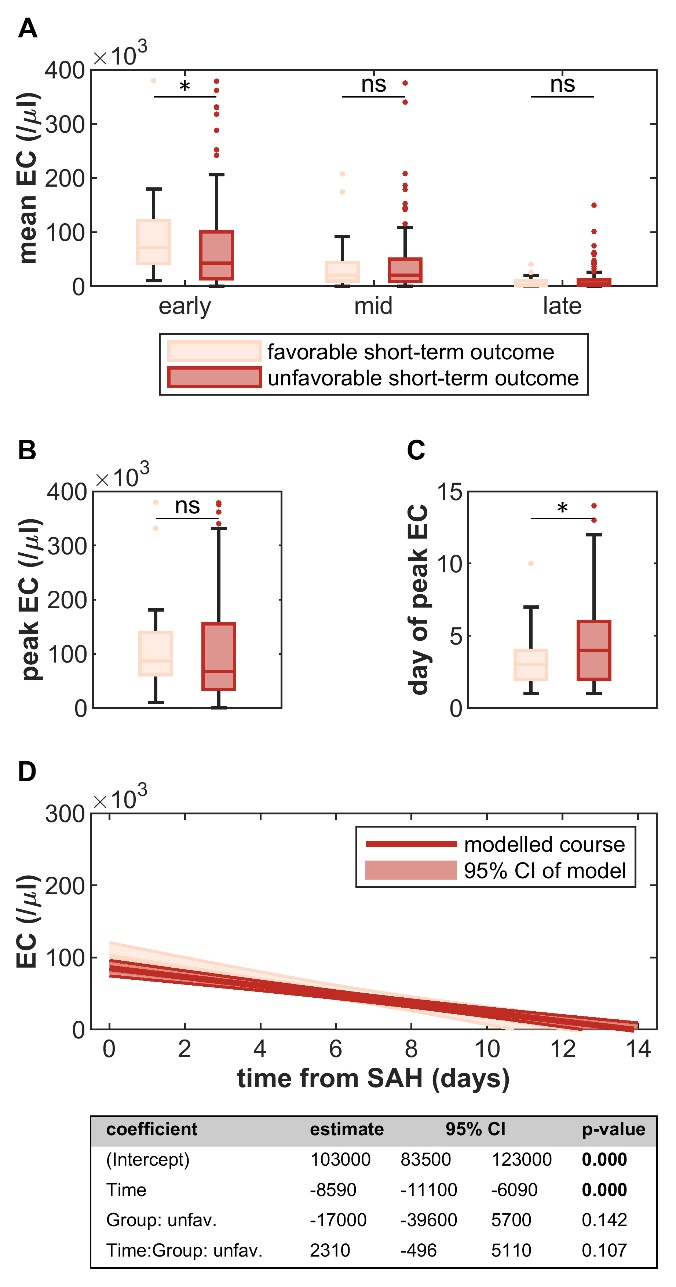
**

**Supplementary Figure 8:** Associations of cerebrospinal fluid (CSF) erythrocyte counts (EC) and short-term functional outcome. **(A)** Comparison of early, mid and late mean EC between patients with favorable and unfavorable outcome. **(B, C)** Comparison of peak EC and day of peak EC between patients with favorable and unfavorable outcome. **(D)** Generalized linear mixed-effects model (GLMEM) of EC decay over time in patients with favorable and unfavorable outcome. Statistical significance: **, p < 0.001; *, p < 0.05; ns, not significant. EC, erythrocyte count; SAH, subarachnoid hemorrhage.

**
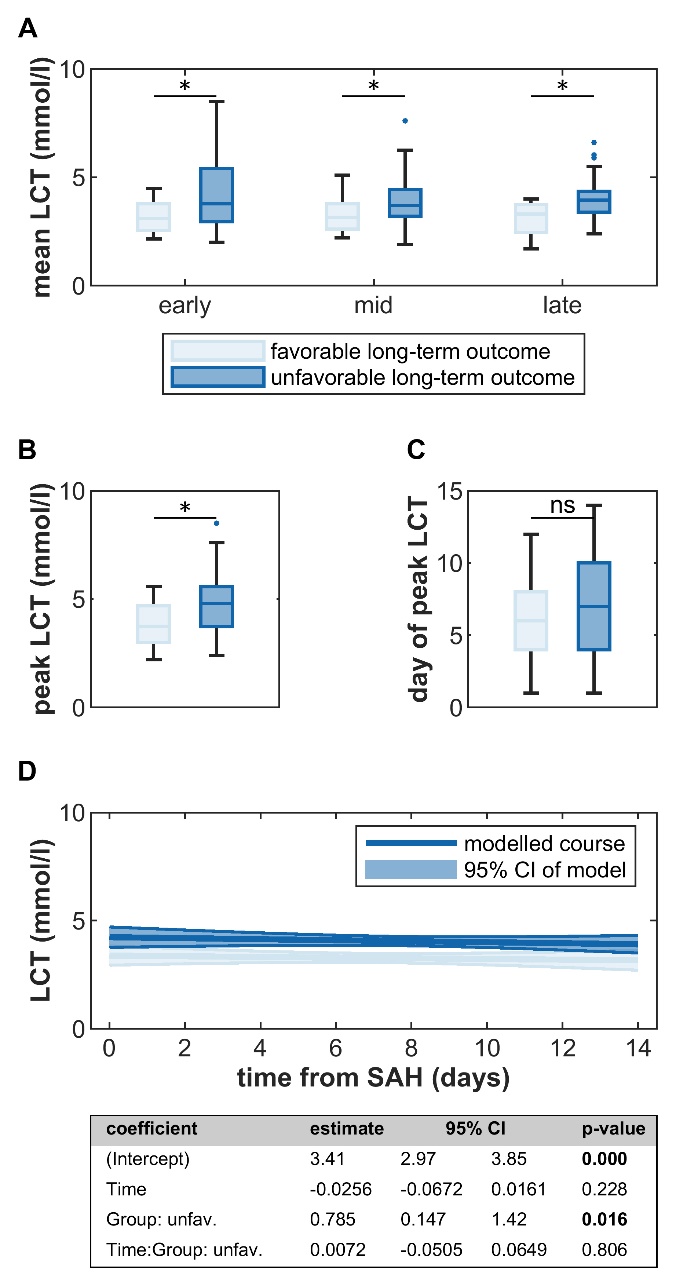
**

**Supplementary Figure 9:** Associations of cerebrospinal fluid (CSF) lactate (LCT) and delayed cerebral ischemia (DCI). **(A)** Comparison of early, mid and late mean LCT between patients without and with DCI. **(B, C)** Comparison of peak LCT and day of peak LCT between patients without and with DCI. **(D)** Generalized linear mixed-effects model (GLMEM) of LCT over time in patients without and with DCI. Statistical significance: **, p < 0.001; *, p < 0.05; ns, not significant. DCI, delayed cerebral ischemia; LCT, cerebrospinal fluid lactate; SAH, subarachnoid hemorrhage.

**
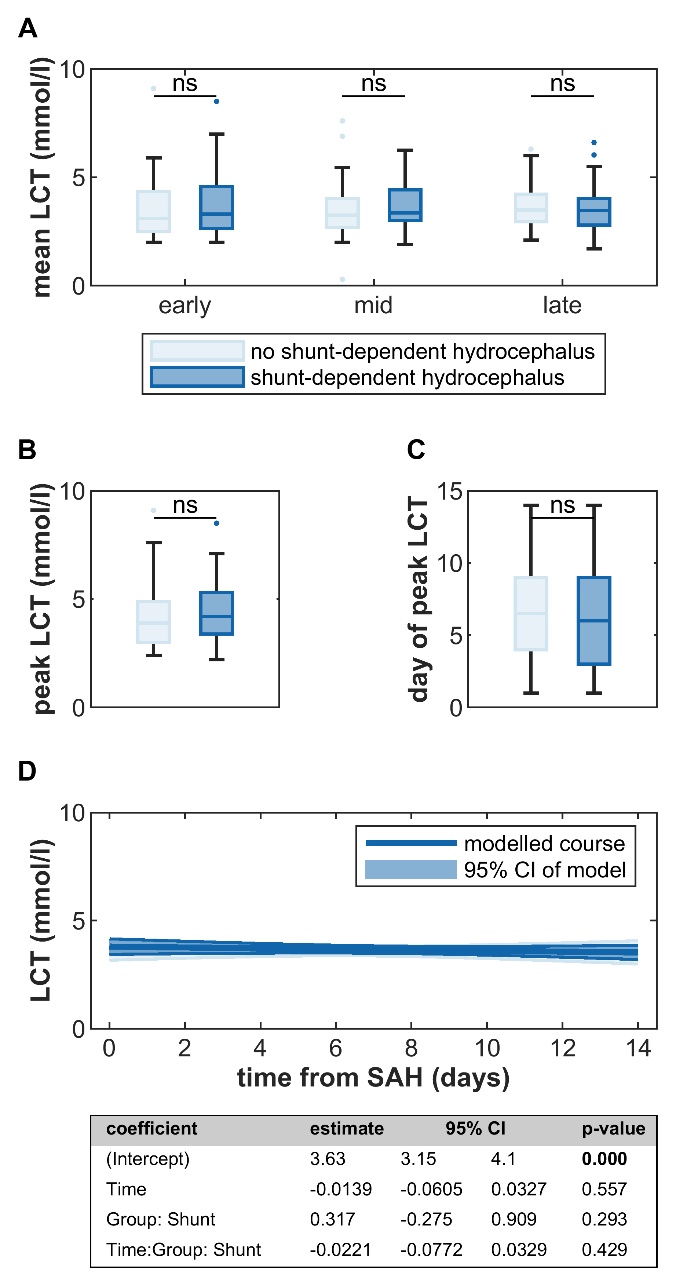
**

**Supplementary Figure 10:** Associations of cerebrospinal fluid (CSF) lactate (LCT) and shunt-dependent hydrocephalus. **(A)** Comparison of early, mid and late mean LCT between patients without and with shunt-dependent hydrocephalus. **(B, C)** Comparison of peak LCT and day of peak LCT between patients without and with shunt-dependent hydrocephalus. **(D)** Generalized linear mixed-effects model (GLMEM) of LCT over time in patients without and with shunt-dependent hydrocephalus. Statistical significance: **, p < 0.001; *, p < 0.05; ns, not significant. LCT, cerebrospinal fluid lactate; SAH, subarachnoid hemorrhage.

**
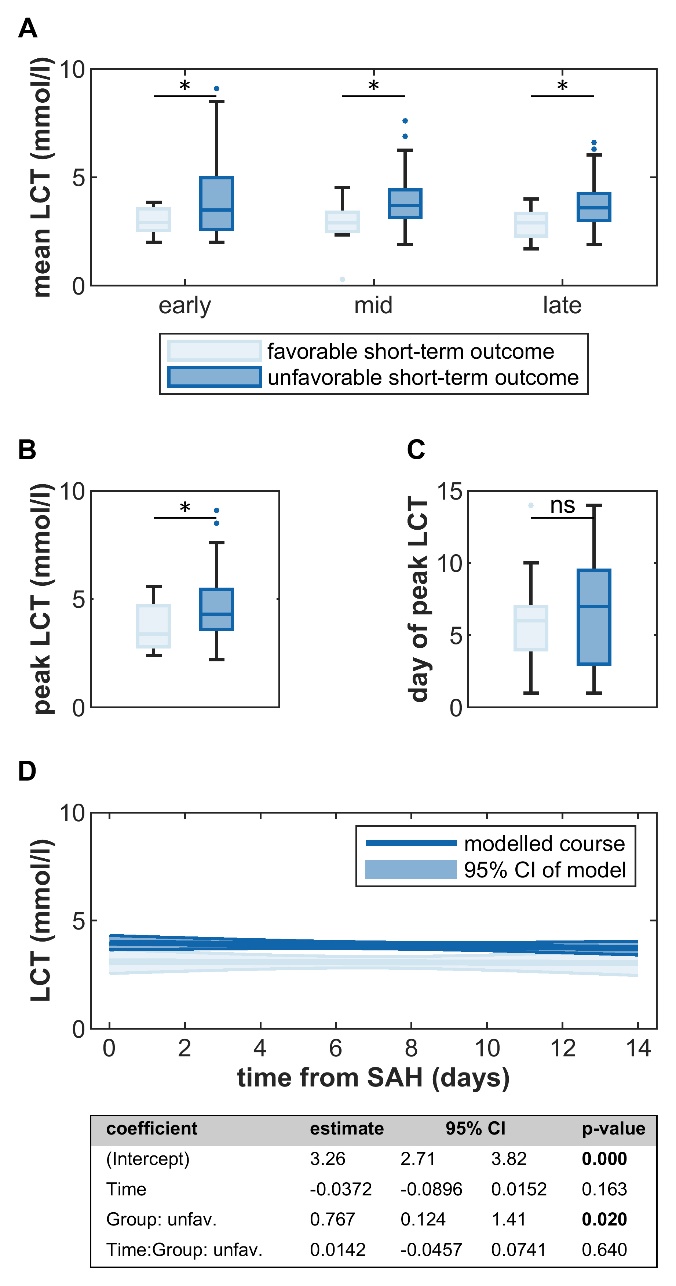
**

**Supplementary Figure 11:** Associations of cerebrospinal fluid (CSF) lactate (LCT) and short-term functional outcome. **(A)** Comparison of early, mid and late mean LCT between patients with favorable and unfavorable outcome. **(B, C)** Comparison of peak LCT and day of peak LCT between patients with favorable and unfavorable outcome. **(D)** Generalized linear mixed-effects model (GLMEM) of LCT over time in patients with favorable and unfavorable outcome. Statistical significance: **, p < 0.001; *, p < 0.05; ns, not significant. LCT, cerebrospinal fluid lactate; SAH, subarachnoid hemorrhage.

**
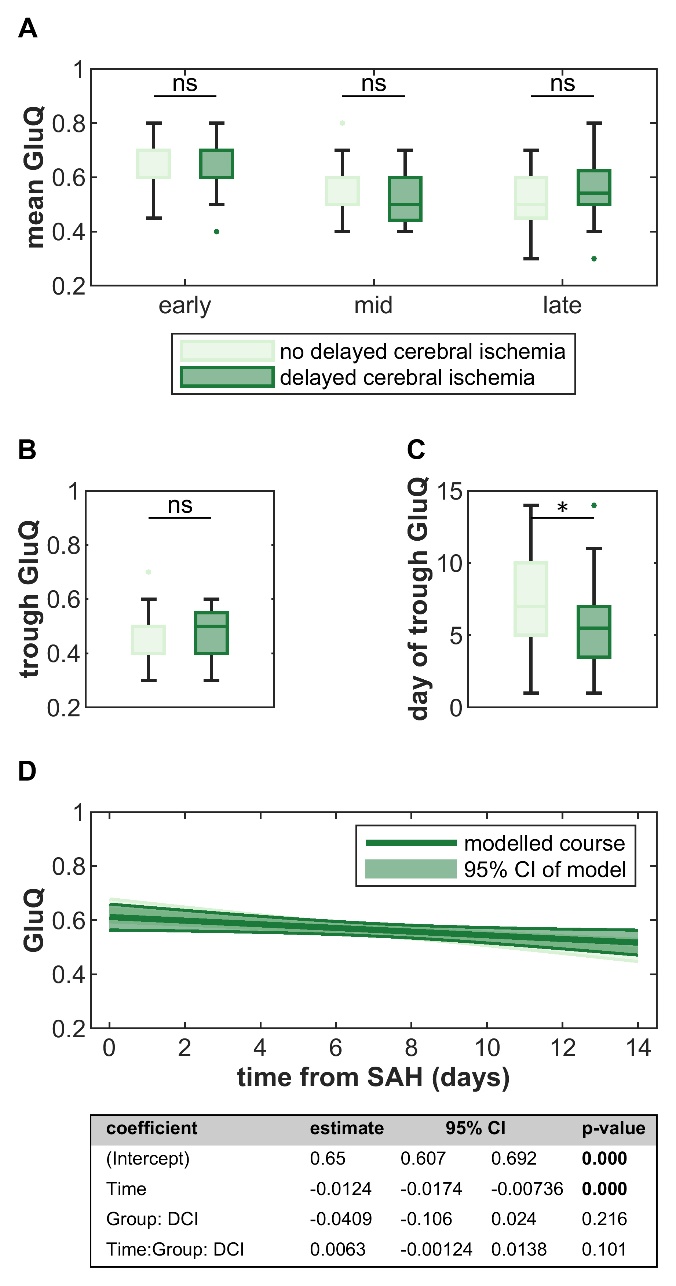
**

**Supplementary Figure 12:** Associations of cerebrospinal fluid (CSF)/plasma glucose ratio (GluQ) and delayed cerebral ischemia (DCI). **(A)** Comparison of early, mid and late mean GluQ between patients without and with DCI. **(B, C)** Comparison of peak GluQ and day of peak GluQ between patients without and with DCI. **(D)** Generalized linear mixed-effects model (GLMEM) of GluQ over time in patients without and with DCI. Statistical significance: **, p < 0.001; *, p < 0.05; ns, not significant. DCI, delayed cerebral ischemia; GluQ, CSF/plasma glucose ratio; SAH, subarachnoid hemorrhage.

**
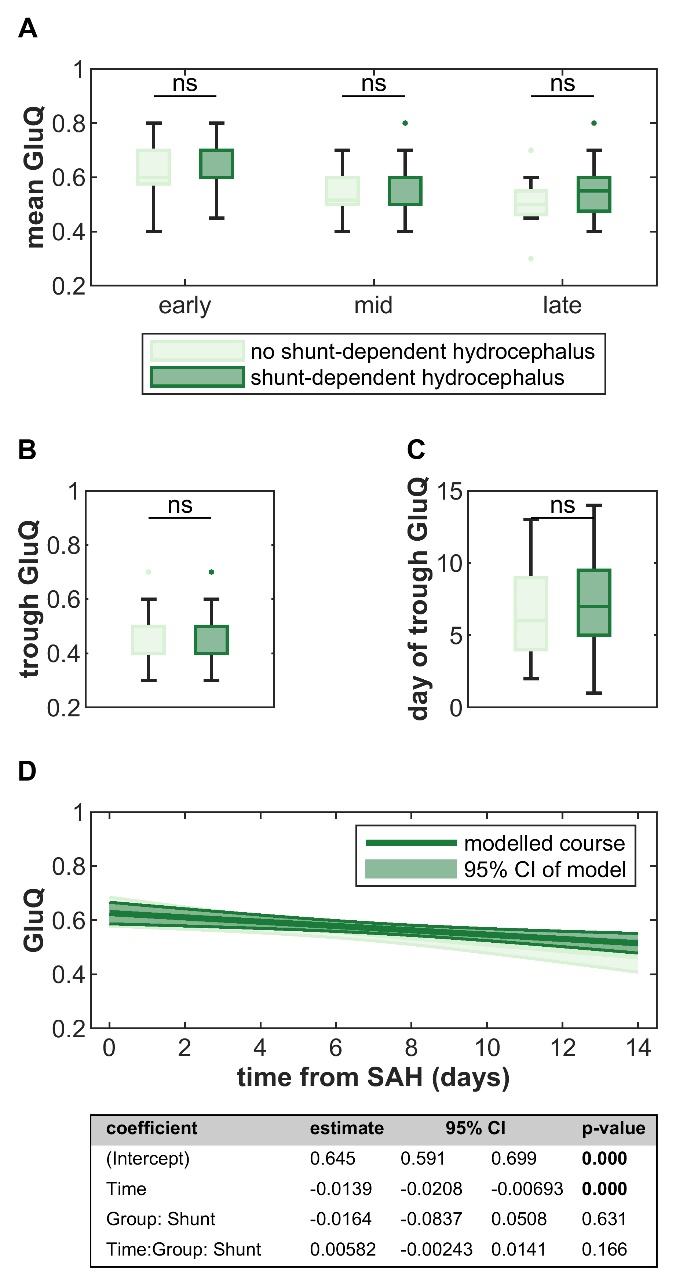
**

**Supplementary Figure 13:** Associations of cerebrospinal fluid (CSF)/plasma glucose ratio (GluQ) and shunt-dependent hydrocephalus. **(A)** Comparison of early, mid and late mean GluQ between patients without and with shunt-dependent hydrocephalus. **(B, C)** Comparison of peak GluQ and day of peak GluQ between patients without and with shunt-dependent hydrocephalus. **(D)** Generalized linear mixed-effects model (GLMEM) of GluQ over time in patients without and with shunt-dependent hydrocephalus. Statistical significance: **, p < 0.001; *, p < 0.05; ns, not significant. GluQ, CSF/plasma glucose ratio; SAH, subarachnoid hemorrhage.

**
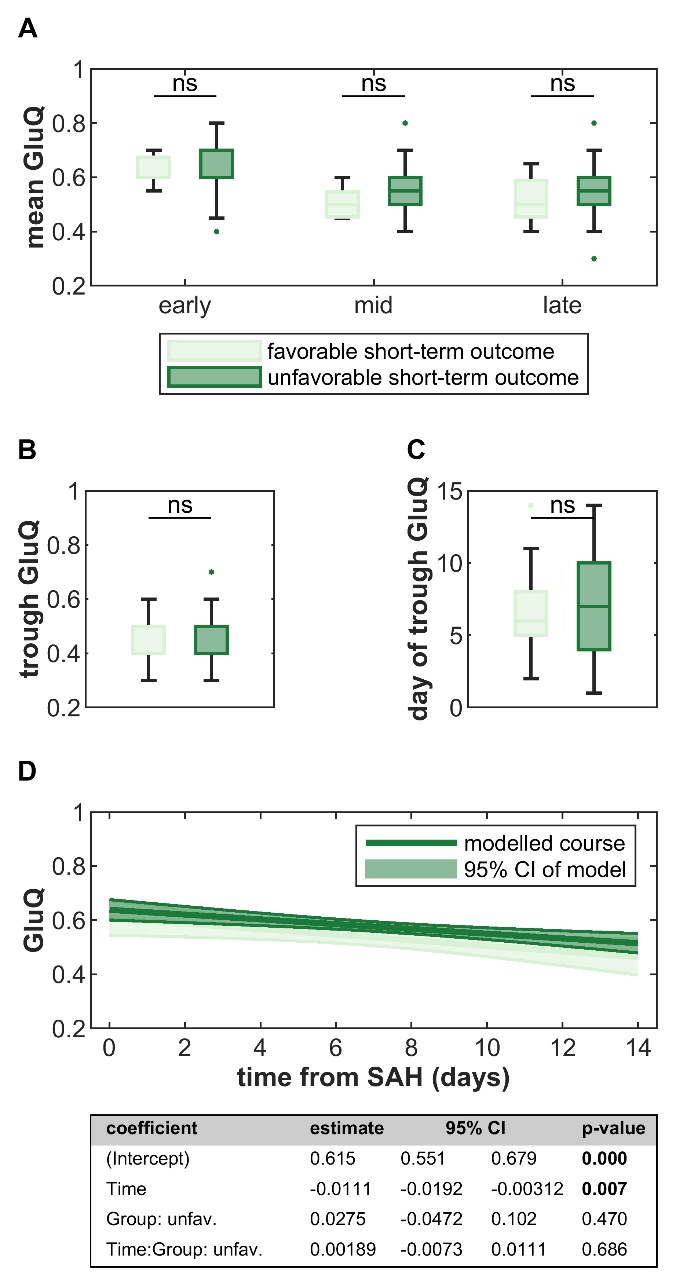
**

**Supplementary Figure 14:** Associations of cerebrospinal fluid (CSF)/plasma glucose ratio (GluQ) and short-term functional outcome. **(A)** Comparison of early, mid and late mean GluQ between patients with favorable and unfavorable outcome. **(B, C)** Comparison of peak GluQ and day of peak GluQ between patients with favorable and unfavorable outcome. **(D)** Generalized linear mixed-effects model (GLMEM) of GluQ over time in patients with favorable and unfavorable outcome. Statistical significance: **, p < 0.001; *, p < 0.05; ns, not significant. GluQ, CSF/plasma glucose ratio; SAH, subarachnoid hemorrhage.

**
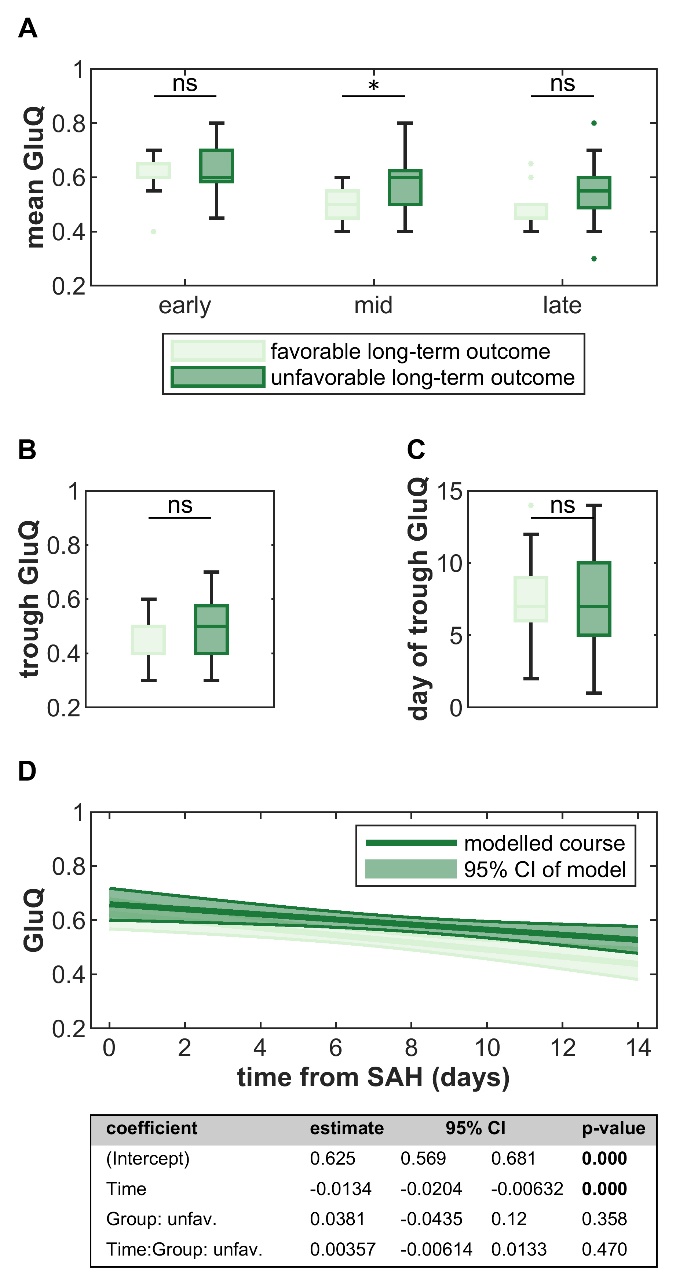
**

**Supplementary Figure 15:** Associations of cerebrospinal fluid (CSF)/plasma glucose ratio (GluQ) and long-term functional outcome. **(A)** Comparison of early, mid and late mean GluQ between patients with favorable and unfavorable outcome. **(B, C)** Comparison of peak GluQ and day of peak GluQ between patients with favorable and unfavorable outcome. **(D)** Generalized linear mixed-effects model (GLMEM) of GluQ over time in patients with favorable and unfavorable outcome. Statistical significance: **, p < 0.001; *, p < 0.05; ns, not significant. GluQ, CSF/plasma glucose ratio; SAH, subarachnoid hemorrhage.

| **ID** | **Age (years)** | **Sex** | **VRI source** | **Diagnosis of VRI** | **Microbe** | **CSF culture** | **CSF PCR** |
| --- | --- | --- | --- | --- | --- | --- | --- |
| 1 | 43 | M | craniectomy wound | 16 days after aSAH | *staphylococcus epidermidis* | positive | not performed |
| 2 | 39 | F | ELD | 24 days after aSAH | *klebsiella pneumoniae* | positive | positive |
| 3 | 53 | F | EVD | 7 days after aSAH | *staphylococcus epidermidis* | positive | not performed |
| 4 | 52 | F | EVD | 3 days after aSAH | *klebsiella pneumoniae* | positive | not performed |
| 5 | 47 | M | EVD | 15 days after aSAH | *staphylococcus epidermidis* | positive | not performed |
| 6 | 31 | M | VPS | 18 days after aSAH | *staphylococcus aureus* | positive | positive |

**Supplementary Table 1:** Patients with confirmed ventriculostomy-related infections (VRI). aSAH, aneurysmal subarachnoid hemorrhage; CSF, cerebrospinal fluid; ELD, external lumbar drain; EVD, external ventricular drain; PCR, polymerase chain reaction; VPS, ventriculoperitoneal shunt.

**
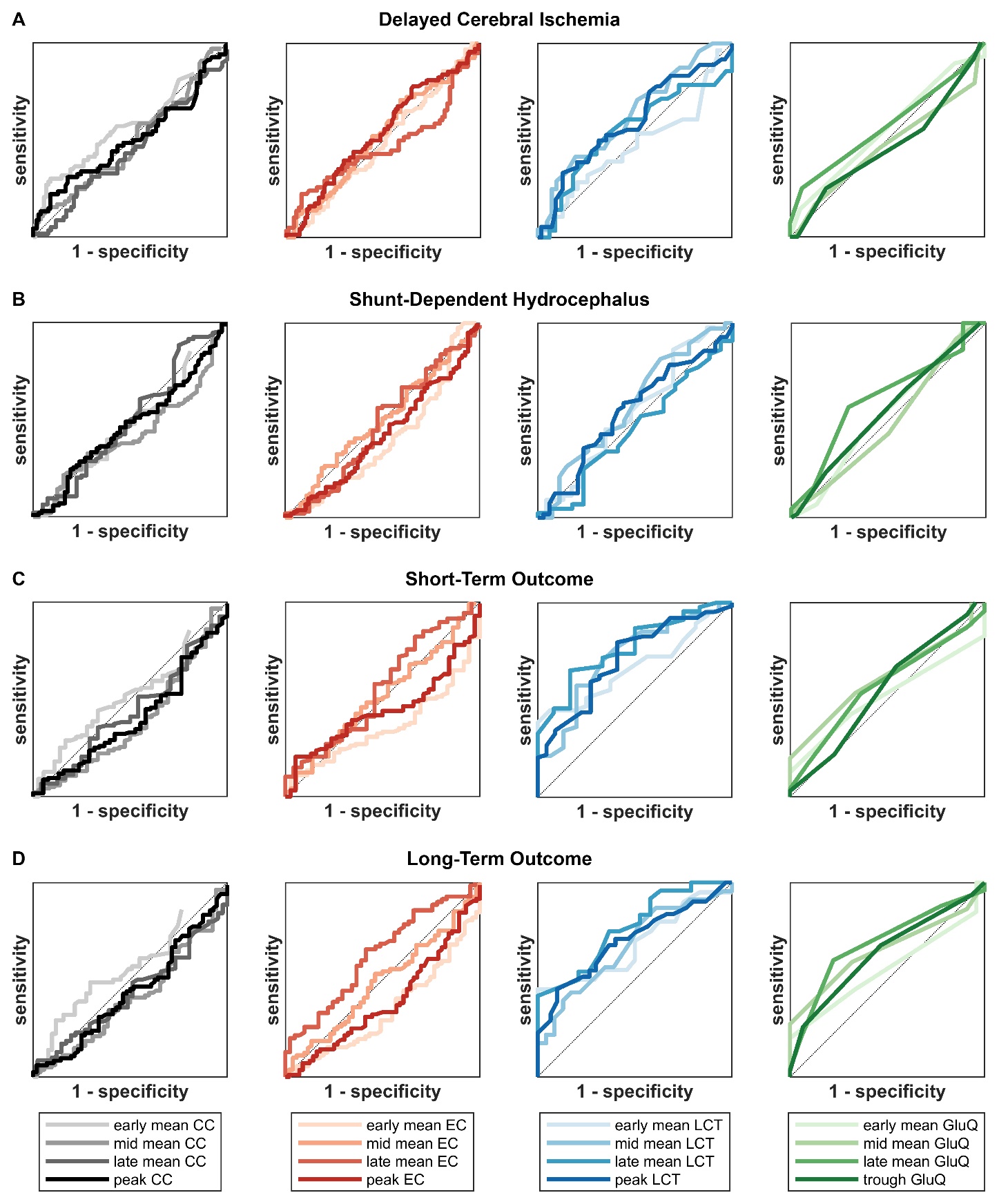
**

**Supplementary Figure 16:** Receiver operating characteristic (ROC) curves of early, mid and late mean and peak or trough values of cerebrospinal fluid (CSF) leukocyte count (CC; black), erythrocyte count (EC; red), lactate (LCT; blue), or CSF/plasma glucose ratio (GluQ; green), respectively, for the identification of **(A)** delayed cerebral ischemia (DCI), **(B)** shunt-dependent hydrocephalus, or **(C)** unfavorable short- and **(D)** long-term functional outcome **(D)**.
